# Olanzapine: Association Between a Typical Antipsychotic Drug and Aortic Calcification

**DOI:** 10.3389/fcvm.2021.710090

**Published:** 2021-09-10

**Authors:** Chao Zhang, Dongdong Zheng, Weijing Feng, Huanji Zhang, Feng Han, Wanbing He, Aiting Liu, Hui Huang, Jie Chen

**Affiliations:** ^1^Department of Cardiology, Sun Yat-sen Memorial Hospital, Sun Yat-sen University, Guangzhou, China; ^2^Department of Psychiatry, Psychiatric Hospital of Guangzhou Civil Affairs Bureau, Guangzhou, China; ^3^Cardiovascular Department, The Eighth Affiliated Hospital, Sun Yat-sen University, Shenzhen, China; ^4^Department of Ultrasound, Cancer Center, Sun Yat-sen University, Guangzhou, China; ^5^Department of Radiation Oncology, Sun Yat-sen Memorial Hospital, Sun Yat-sen University, Guangzhou, China

**Keywords:** aortic calcification, mental illness, schizophrenia, olanzapine, antipsychotic drugs

## Abstract

**Aims:** This study concentrates on the relationship between antipsychotic drugs (APDs) and aortic calcification.

**Methods:** All 56 patients with schizophrenia were divided into two groups according to aortic calcification index. APD equivalent dose was calculated via defined daily doses method.

**Results:** In schizophrenia patients with higher aortic calcification index scores, APD equivalent doses were lower. APD equivalent dose was negatively related to aortic calcification index. Although equivalent APD dose in patients without olanzapine treatment was negatively related to aortic calcification index, it seems that equivalent APD dose did not associate with aortic calcification.

**Conclusion:** Aortic calcification is negatively associated with APD dose in schizophrenia patients. Olanzapine seems to be vital to the relationship between aortic calcification and APD treatment.

## Introduction

Severe mental illness has persistent procession, and schizophrenia causes serious disabilities to individuals worldwide (1). Antipsychotic drugs (APDs) are essential in providing recovery opportunities for schizophrenia patients (2, 3). However, besides alleviating symptoms, APDs also lead to adverse effects, especially elevating cardiovascular risks (4).

Several APDs have been proven to have metabolic effects, which would cause cardiovascular diseases (5). Jess Fiedorowicz et al. reported that vasculopathy is related to psychosis and arterial stiffness is also enhanced in patients with conventional APD exposure (6). Different APDs exert adverse side effects on the vascular system, based on their metabolic effects (7). Olanzapine has been confirmed to result in metabolic syndromes and exert several adverse cardiovascular effects (8, 9). Olanzapine has also been widely used in cancer patients to avoid chemotherapy-caused nausea and vomiting (10). Of note, the dose of APD is especially vital for all patients who underwent such a medication process (10).

Emerging evidence suggests that APD dose should be personalized and cardiovascular risk must be taken into consideration (11). Aortic calcification is acknowledged as a vital predictor of cardiovascular risk (12). Moreover, olanzapine could reduce blood pressure and cardiac contractile function *in vivo* (8). However, the relationship between aortic calcification and APD treatment, especially olanzapine, was not clear. In this study, we investigated schizophrenia patients to explore the relationship between APD treatment and aortic calcification.

## Methods

### Study Subjects and Clinical Information Extraction

Between December 2016 and August 2017, 79 patients diagnosed with schizophrenia were enrolled in this study. All these patients sought routine follow-ups at the Psychiatric Hospital of Guangzhou Civil Affairs Bureau. We enrolled patients of both sexes, and both the patients and their relatives consented to our accessing their medical records. The study was approved by the Ethics Committee of Psychiatric Hospital of Guangzhou Civil Affairs Bureau. The methods complied with the ethical guidelines of the 1975 Declaration of Helsinki. All the subjects, or their agents, provided their written informed consent.

Diagnosis of schizophrenia was conducted according to Chinese Classification and Diagnosis of Mental Diseases and confirmed by at least two different independent psychiatrists (13). All the subjects enrolled in this study underwent a continuous oral APD treatment for at least 1 year. Patients who received more than one kind of APD treatment or who were admitted to a hospital for further treatment were excluded. The patients' APD doses were unchanged for at least 6 months. In total, 56 patients with schizophrenia were enrolled in this study. Clinical information including blood sample measurements was obtained in the medical records, and individual histories were provided by patients or their relatives. In addition, no interference is present in this manuscript, and written informed consent was provided by the patients or their guardians.

### Collection of Blood Samples and Baseline Diameters Measurement

Blood samples were collected and sent to the clinical laboratory of the Psychiatric Hospital of Guangzhou Civil Affairs Bureau. Some biochemical characteristics including lipoprotein metabolism, hepatic function, and blood routines were measured by a standardized procedure.

Blood pressure and body mass index (BMI) were measured and calculated by at least 2 nurses from the clinic department and confirmed by their relatives. ECGs were conducted and diagnosed by the ECG section and confirmed by physicians.

### Aortic Calcification Index Calculation

Aortic calcification (AoAC) indices were obtained via chest X-rays. The chest radiology information was obtained from the medical imaging department of Psychiatric Hospital of Guangzhou Civil Affairs Bureau. The aortic calcification index was calculated by experienced radiologists using established methods (14). Briefly, each plain chest radiography was divided into 16 sections according to the aortic arch and assigned a calcification index present as a percentage in our study. The aortic calcification indices were graded on a scale from 0 to 3, as in previous studies (15, 16).

### APD Equivalent Dose Calculation

According to the defined method for minimum effective dose, APD doses were standardized into equivalent doses for further analysis (17). APD equivalent dose was calculated by the defined daily doses (DDD) method (18). The standardization process was based on chlorpromazine, and the duration of APD treatment was recorded in years. The accumulated APD treatment was calculated by multiplying the standardized daily dose by the duration of the treatment. Accumulated APD treatment was tested for the normal distribution. Log transformation was conducted if necessary.

### Statistical Analysis

All such data extracted from medical records are presented in this article, with continuous data as mean values with a standard deviation (SD) and categorical data as frequencies with percentages. Before comparison, all continuous data underwent non-parametric tests to confirm normal distribution. For those normal distribution values, comparisons were conducted by Student's *t*-test and multiple regression analysis. Some of the non-normal data were transformed, which was further confirmed by nonparametric tests, (specific details are included in the results section). Nonparametric comparisons were conducted on the data that could not be transformed. Furthermore, Pearson's correlations were conducted for parametric data and Spearman's correlations were conducted for non-parametric data, referred to as r or r_S_. To reveal the independent factors, multiple regression analysis was used. All statistical analysis was performed using the software SPSS 20.0. For all statistical tests, two-tailed *p*-values < 0.05 was the threshold of statistical significance.

## Results

### Comparison of Aortic Calcification, Accumulated APD Treatment, and Clinical Characteristics in Schizophrenia Patients Receiving APDs

**Figure 1:**
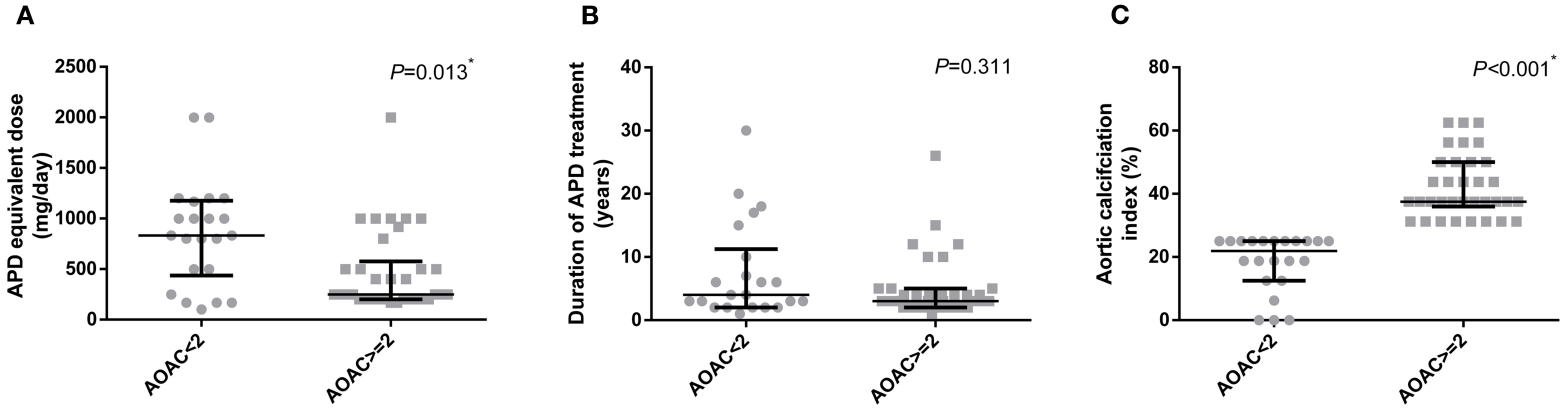
Comparison of APD medication profiles in patients according to their AoAC index. **(A)** APD equivalent dose, **(B)** duration of APD treatment, **(C)** Aortic calcification index. *p < 0.05. APD, antipsychotic drug.

All schizophrenia patients included in this project were divided into two groups according to their AoAC score. The comparison of clinical characteristics and kinds of ADP treatment are shown in Table 1. It is shown that patients with higher AoAC scores were older, male, and inclined to develop smoking habits. Comparison of APD profiles indicated that in the high AoAC score group, APD equivalent doses were lower, with no difference in the duration of APD treatment (Figure 1).

**Table 1:** Comparation between patients with different severities of aortic calcification.

	**AoAC < 2**	**AoAC ≥ 2**	***P***
Age (years)	56.09 ± 12.35	65.08 ± 8.00	0.001^*^
Gender (Male%)	3 (13.6%)	16 (47.1%)	0.010^*^
Smoking (*n*%)	0 (0%)	9 (26.5%)	0.008^*^
Hypertension (*n*%)	6 (27.3%)	11 (32.4%)	0.686
DM (*n*%)	4 (18.2%)	11 (32.4)	0.242
CAD (*n*%)	1 (4.5%)	7 (20.6%)	0.130
Stroke (*n*%)	2 (9.1%)	2 (5.9%)	0.642
BMI (kg/m^2^)	22.74 ± 3.48	21.40 ± 3.12	0.146
WBC (10^9^/L)	7.41 ± 1.80	7.17 ± 1.73	0.605
RBC (10^9^/L)	4.04 ± 0.48	3.92 ± 0.66	0.467
PLT (10^9^/L)	267.36 ± 90.97	251.71 ± 93.42	0.539
ALT (U/L)	18.05 ± 6.90	20.29 ± 9.01	0.324
AST (U/L)	20.05 ± 6.86	21.56 ± 5.94	0.385
γ-GGT (U/L)	22.23 ± 14.65	21.18 ± 10.54	0.756
TG (mmol/L)	1.54 ± 0.94	1.49 ± 0.87	0.845
TC (mmol/L)	4.70 ± 0.90	4.79 ± 1.06	0.765
Glucose (mmol/L)	5.39 ± 0.99	5.78 ± 2.33	0.395

*ALT, alanine aminotransferase; APD, antipsychotic drugs; AST, aspartate aminotransferase; BMI, body mass index; CAD, coronary artery disease; DM, diabetes mellitus; TC, total cholesterol; TG, triglyceride; γ-GGT, γ glutamyl transferase*.

* *P < 0.05*.

### Relationship Between Aortic Calcification Index, APD Treatment, and Clinical Characteristics

To further investigate the relationship between the aortic calcification index and other potential factors, Spearman's correlation analysis was conducted. As shown in Table 2, aortic calcification index was related to age, sex, and smoking (*r* = 0.536, *P* < 0.001; *r* = 0.332, *P* = 0.012; *r* = 0.349, *P* = 0.008). In addition, APD equivalent dose was negatively related to aortic calcification index (*r* = −0.413, *P* = 0.002). It is indicated that APD equivalent dose would be the one of factors for aortic calcification.

**Table 2:** Relationship between aortic calcification index and clinical characteristics.

	**r_**s**_**	***P***
Age	0.536	<0.001^*^
Gender	0.332	0.012^*^
Smoking	0.349	0.008^*^
Hypertension	0.148	0.276
DM	0.102	0.454
CAD	0.207	0.125
Stroke	0.035	0.800
BMI	−0.235	0.082
WBC	−0.017	0.900
RBC	−0.202	0.136
PLT	0.036	0.794
ALT	0.175	0.197
AST	0.140	0.304
γ-GGT	0.033	0.811
TG	−0.039	0.773
TC	−0.122	0.368
Glucose	−0.159	0.243
APD equivalent dose	−0.413	0.002^*^
Duration of APD treatment	−0.151	0.266

*ALT, alanine aminotransferase; APD, antipsychotic drugs; AST, aspartate aminotransferase; BMI, body mass index; CAD, coronary artery disease; DM, diabetes mellitus; TC, total cholesterol; TG, triglyceride; γ-GGT, γ glutamyl transferase*.

* *P < 0.05*.

### Olanzapine Treatment and Aortic Calcification in Schizophrenia Patients

**Figure 2:**
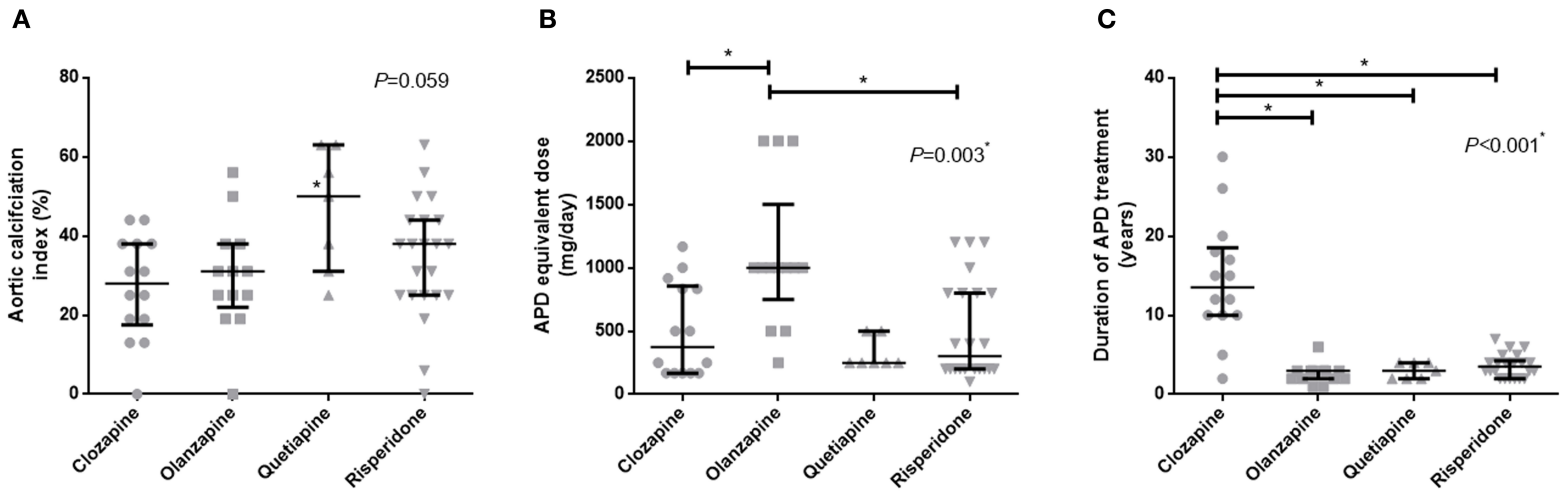
Comparison of aortic calcification index and APD treatment in patients with different kinds of APDs. **(A)** APD equivalent dose, **(B)** duration of APD treatment, **(C)** Aortic calcification index. *p < 0.05. APD, antipsychotic drugs.

Patients were divided into four groups, based on which APD was used in their treatment. It is shown in Figure 2 that the aortic calcification indices in these groups (clozapine, olanzapine, quetiapine, and risperidone) did not differ. However, the duration and equivalent dose were quite different between groups, and olanzapine treated patients seemed to have higher APD equivalent doses compared to the clozapine and quetiapine groups (Figure 2B). Although clozapine-treated patients had longer durations of APD treatment, it seems that the duration of APD treatment was not related to aortic calcification, as shown in Table 2.

**Figure 3:**
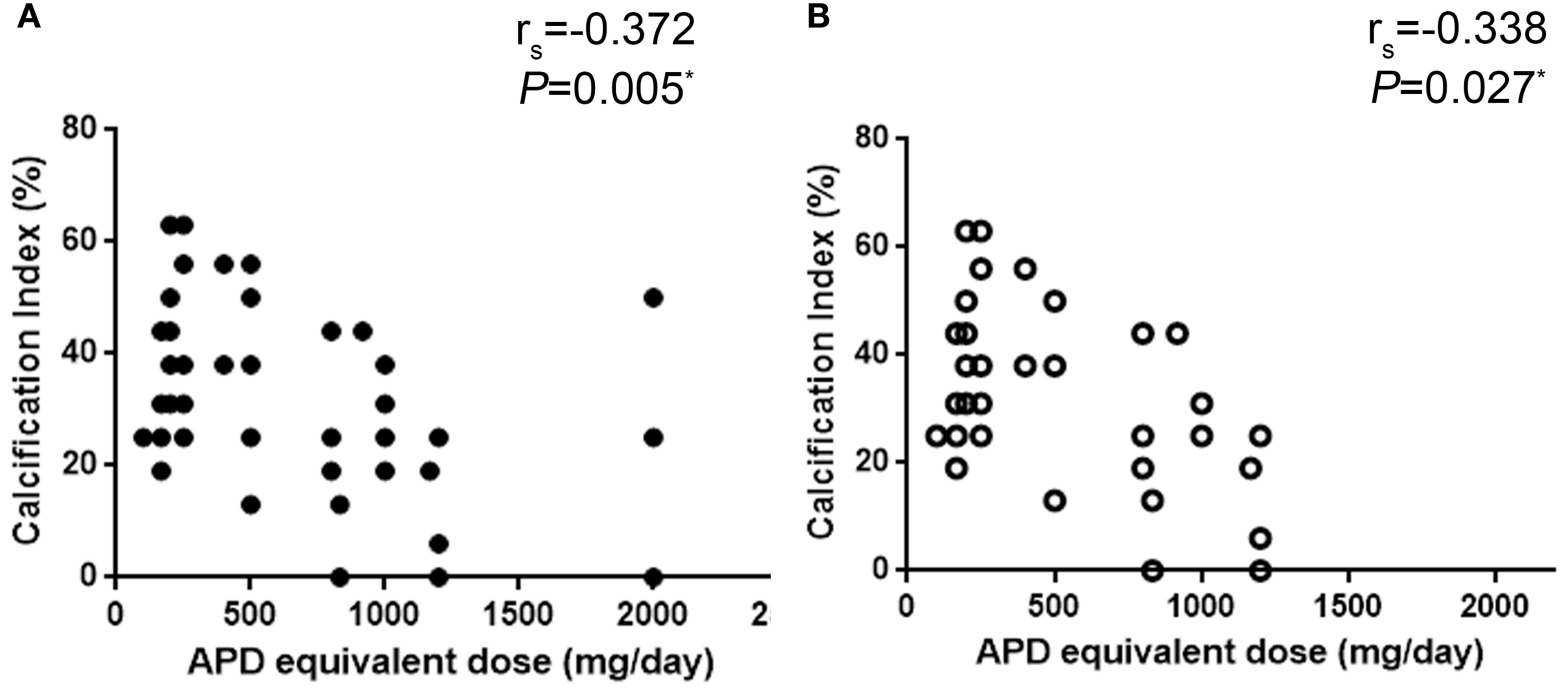
Correlation analysis of equivalent APD dose and aortic calcification index. **(A)** all patient, **(B)** patients without olanzapine treatment. APD, antipsychotic drugs.

Correlation analysis showed that in patients who were not treated with olanzapine, the aortic calcification index was associated with APD equivalent dose (*r* = −0.338, *P* = 0.027, Figure 3). Considering the limited number of patients in each group, and that age is a major factor in the development of aortic calcification, age should be ruled out while comparing equivalent APD dose and aortic calcification index. Ordinal regression analysis indicated that in all patients age and equivalent APD dose are vital factors associated with aggravation of aortic calcification (age: OR = 1.122, *P* < 0.001; equivalent APD dose: OR = 0.999, *P* = 0.021). However, in patients without olanzapine treatment, only age is associated with aggravation of aortic calcification (OR = 1.126, *P* = 0.001) (Table 3). Such results indicated that olanzapine would be a vital factor related to aortic calcification, regardless of age.

**Table 3:** Ordinal regression analysis between AoAC grade and potential factors in patients with APD treatment or without olanzapine treatment.

**All patients**	**OR**	**95% CI**	***P***
Age	1.122	(1.054, 1.194)	<0.001^*^
APD equivalent dose	0.999	(0.997, 1.000)	0.021^*^
Gender (Female)	0.404	(0.096, 1.700)	0.216
Gender (Male)	1	–	–
Smoking	0.586	(0.091, 3.759)	0.573
Non-smoking	1	–	–
**Not Olanzapine**	**OR**	**95% CI**	***P***
Age	1.126	(1.048, 1.210)	0.001^*^
APD equivalent dose	0.998	(0.996, 1.000)	0.087
Gender (Female)	0.312	(0.045, 2.147)	0.237
Gender (Male)	1	–	–
Smoking	1.668	(0.156, 17.880)	0.673
Non-smoking	1	–	–

*APD, antipsychotic drugs*.

* *P < 0.05*.

## Discussion

Using an aortic calcification indices from chest X-rays is believed to be one most available and effective methods to predict cardiovascular events (19). To describe the severity of aortic calcification degree, AoAC score was used to predict the cardiovascular risks (15). Although calcification evaluation from chest X-ray might not be as accurate as from CT (20), long-term APD treatment for schizophrenia patients tend to suffer more from financial burdens (21), and it is quite hard for patients or caregivers to arrange such examinations. In our study, schizophrenia patients were enrolled and APD equivalent dose was higher in the group with AoAC ≥ 2, indicating that some potential relationship between aortic calcification and APD treatment. Some recent studies revealed that besides some potential benefits, long-term APD treatment did indeed lead to several adverse results in patients' cardiovascular and metabolic systems, including disrupting lipid and glucose metabolism (22).

Presently, APD treatment is still a major beneficial treatment for schizophrenia patients that decreases the risk of relapse and reduces all-cause mortality (23). Emerging evidence indicated that APDs could also contribute to overall 5-year diabetes occurrence (24). Some pharmacological research also emphasized that long-term use of APD treatment would leave schizophrenia patients suffering from adverse effects, including cardiovascular risks and brain structure changes (25). As one efficient method to evaluate cardiovascular risk, aortic calcification was proven to be negatively related to equivalent APD dose, suggesting that APD exerts some potential effects on patients (Table 2). Despite this, schizophrenia patients with olanzapine treatment suffered more from cardiovascular risk, and the major disturbance from olanzapine is based on metabolic abnormalities (26).

Due to the high heterogeneity of atypical APDs, different kinds of APDs exert various effects, especially on the cardiovascular system (27). Of note, olanzapine seems to dramatically enhance cardiovascular risk compared to all other atypical APDs, including risperidone, clozapine, and quetiapine (22). Surprisingly, this study showed that the aortic calcification index is negatively associated with APD dose increment. Olanzapine has a higher equivalent dose among patients, and in patients without olanzapine treatment the relationship between equivalent APD dose and aortic calcification index vanishes. Although olanzapine is proven to induce some metabolic disorders (9), it is reported recently that olanzapine might have some extra protective effects such as reducing reactive oxygen species induced cell death (28). Therefore, the exact mechanism of olanzapine in the pathological process of aortic calcification needs further investigation.

Our study had several limitations. Firstly, the number of patients involved in our study was relatively small. Due to the poor management of schizophrenia patients in China, long-term APD treatments and monitoring were not satisfying, and some schizophrenia patients and their guardians or relatives refused to do further tests in clinics. Secondly, the present work is based on one sectional study and we could only reveal the relationship between aortic calcification and APD equivalent dose. We could not rule out the possibility that patients with lower aortic calcification could tolerate higher doses of APD. Thus, further studies with larger sample sizes and cohort studies are needed to confirm our findings.

## Conclusions

It is shown in this study that aortic calcification was negatively correlated with APD equivalent dose in schizophrenia patients, and that olanzapine plays a role in aortic calcification. The marked effects of APD treatment on aortic calcification should not be ignored when choosing different kinds of APDs.

## Data Availability Statement

The raw data supporting the conclusions of this article will be made available by the authors, without undue reservation.

## Ethics Statement

Written informed consent was obtained from the individual(s) for the publication of any potentially identifiable images or data included in this article.

## Author Contributions

CZ, DZ, WF, and HH were responsible for study design, statistical analysis, and manuscript preparation. CZ, DZ, WF, FH, WH, HZ, AL, JC, and HH were responsible for subjects recruitment and clinical data collecting. CZ, WF, JC, and HH were involved in writing the protocol and providing the funding for this study. All authors contributed to and have approved the final manuscript.

## Funding

This work was supported in part by National Natural Science Foundation of China (NSFC) (82073408 and 81500563) to JC, Funds for International Cooperation and Exchange of NSFC, NSFC-FDCT (8201101103), NSFC (81870506 and 81670676), Guangzhou Science and Technology Plan Project (201607010075) and Futian District Public Health Scientific Research Project of Shenzhen (FTWS2019003) to HH.

## Conflict of Interest

The authors declare that the research was conducted in the absence of any commercial or financial relationships that could be construed as a potential conflict of interest.

## Publisher's Note

All claims expressed in this article are solely those of the authors and do not necessarily represent those of their affiliated organizations, or those of the publisher, the editors and the reviewers. Any product that may be evaluated in this article, or claim that may be made by its manufacturer, is not guaranteed or endorsed by the publisher.
